# Emergency Psychiatry in adolescence and emerging adulthood: A three-year retrospective study

**DOI:** 10.1192/j.eurpsy.2026.10936

**Published:** 2026-07-31

**Authors:** C. Mauceri, C. Paz Eraso, F. Gavelli, M. Bellan, E. Gambaro, C. Gramaglia, P. Zeppegno

**Affiliations:** 1 Department of Translational Medicine, Università del Piemonte Orientale; 2 Emergency Medicine Department, Maggiore della Carità University Hospital; 3 Psychiatry Unit, Maggiore della Carità Hospital, Department of Translational Medicine, Università del Piemonte Orientale, Novara, Italy

## Abstract

**Introduction:**

Recently, there has been a significant increase in emergency department (ED) admissions among adolescents and young adults seeking help for mental health–related concerns worldwide (Bommersbach et al. JAMA 2023). Thus, the ED might have a potential role in the early detection and timely intervention during the vulnerable adolescent transition period to “emerging adulthood”, in order to optimize care pathways and clinical outcomes (Brdar et al. JCPP Adv 2025).

**Objectives:**

All patients aged ≤25 years admitted to the adult Emergency Department (AED) for psychiatric evaluation (PE) at “Maggiore della Carità” University Hospital in Novara, Italy, between 2021 and 2024 were included.

**Methods:**

Socio-demographic and clinical characteristics, reasons for referral, psychiatric diagnoses at discharge, and outcomes of psychiatric ED consultation were retrospectively analyzed.

**Results:**

Out of total 1856 AED admissions, 366 (19.7%) underwent a PE. The mean age was 21.2 years (SD = 2.25; range 16–25 years), of whom 25 (6.8%) were minors (<18 years). The majority of patients were female and Italian (n = 196, 81.2%). Overall, a significant upward trend of PE, corresponding to an average annual increase of approximately 20% (p < 0.01), was found. Positive psychiatric history (72.9%) with prior documented hospitalizations (42.5%) was observed while a previous contact with addiction services was reported in fewer cases (7.3%). Reasons for consultation were anxiety (38.7%), suicidal ideation (30.2%), psychomotor agitation not substance-related (15.9%), mood and psychotic symptoms (10.2–9.6%) and intoxication (7.2%). According to DSM-5, psychiatric diagnosis was assessed in 76.8% of cases: personality disorders (33.2%), anxiety disorders (15.4%) and psychotic disorders (11.9%) were mostly reported. Substance-related findings included positive alcohol tests (5.6%) and urine screening positivity for cannabinoids (45.5%), benzodiazepines (64.2%), cocaine (13.8%) and amphetamines (3.3%). Main intervention outcomes were voluntary hospitalization (35.2%), referral to outpatient services (23.9%) and discharge (19.1%).

**Conclusions:**

This study shows the increasing number of PE in emergency setting among adolescents and youths, in line with current literature. Both the high prevalence of psychiatric diagnoses and the frequent reports of suicidal ideation and psychomotor agitation emphasize the need for structured evaluation protocols and tailored intervention for acute conditions and management of chronic disorders in this age group

**Disclosure of Interest:**

None Declared

